# Within-Plant Bottom-Up Effects Mediate Non-Consumptive Impacts of Top-Down Control of Soybean Aphids

**DOI:** 10.1371/journal.pone.0056394

**Published:** 2013-02-19

**Authors:** Alejandro C. Costamagna, Brian P. McCornack, David W. Ragsdale

**Affiliations:** 1 Department of Entomology, University of Minnesota, St. Paul, Minnesota, United States of America; University of California, Berkeley, United States of America

## Abstract

There is increasing evidence that top-down controls have strong non-consumptive effects on herbivore populations. However, little is known about how these non-consumptive effects relate to bottom-up influences. Using a series of field trials, we tested how changes in top-down and bottom-up controls at the within-plant scale interact to increase herbivore suppression. In the first experiment, we manipulated access of natural populations of predators (primarily lady beetles) to controlled numbers of *A. glycines* on upper (i.e. vigorous-growing) versus lower (i.e. slow-growing) soybean nodes and under contrasting plant ages. In a second experiment, we measured aphid dispersion in response to predation. Bottom-up and top-down controls had additive effects on *A. glycines* population growth. Plant age and within-plant quality had significant bottom-up effects on aphid size and population growth. However, top-down control was the dominant force suppressing aphid population growth, and completely counteracted bottom-up effects at the plant and within-plant scales. The intensity of predation was higher on upper than lower soybean nodes, and resulted in a non-consumptive reduction in aphid population growth because most of the surviving aphids were located on lower plant nodes, where rates of increase were reduced. No effects of predation on aphid dispersal among plants were detected, suggesting an absence of predator avoidance behavior by *A. glycines*. Our results revealed significant non-consumptive predator impacts on aphids due to the asymmetric intensity of predation at the within-plant scale, suggesting that low numbers of predators are highly effective at suppressing aphid populations.

## Introduction

Herbivores are regulated by a combination of top-down and bottom-up forces, but the relative strengths of these forces vary among communities [1]–[3]. The plant vigor hypothesis proposed that plant modules growing vigorously result in higher herbivore abundance [4]. Recent reviews have shown that this hypothesis is supported for most insect herbivores, including sap-sucking insects [5]. However, most studies focused on gall-making insects, which are strong “flush feeders” that need to feed on growing plant tissue in order to develop their galls, and were the original inspiration for the hypothesis [4]–[6]. Thus, despite the importance of this mechanism, the contribution of plant vigor as a bottom-up factor in regulating herbivores, and in particular its interaction with top-down forces, have not been often studied for non-gall-making insects.

Top-down control of herbivores is usually attributed to consumption by predators [7]. However, there is increasing evidence that non-consumptive impacts of predation can also result in strong prey suppression that cascades down to the next trophic level [8]–[10]. Some of these non-consumptive impacts include trait-mediated effects of predators on the behavior [7], [11], [12], morphology [13], or life history parameters of prey [9]. Other types of non-consumptive, negative predator impacts involve predatory behaviors rather than prey-specific attributes, including preference for prey of larger size [14] or prey located on higher quality host plants [15]. Despite the increasing amount of evidence on the importance of non-consumptive impacts of top-down controls on herbivores, few studies have experimentally tested them in combination with bottom-up controls (e.g. [15]).

Although progress has been made in investigating the relative strength of top-down and bottom-up controls on herbivores, few studies have used manipulative experiments in agroecosystems (but see [2], [16], [17]). Such studies in agroecosystems have the advantage that the uniformity of crop habitats facilitates replication. Furthermore, they may inform pest management, because successful biological control is achieved in a top-down manner, and increased plant yield is achieved via cascading effects to the plant level [18]. In previous studies we demonstrated a shift in the within-plant distribution of the soybean aphid, *Aphis glycines* Matsumura (Hemiptera: Aphididae) towards more mature leaves in response to predation [19]. Population increase of other aphid species on slow-growing plant parts is lower than on vigorous-growing plant parts [20]–[22], as predicted by the plant vigor hypothesis [4], although this is unknown for *A. glycines*. Therefore, we hypothesize that 1) vigorous-growing plant parts support higher *A. glycines* population growth rates, and 2) predation on vigorous-growing plant parts results in non-consumptive reductions in *A. glycines* population growth rates by differentially removing individuals with the highest reproductive potential. Here we report on field manipulations of predator access to the upper (i.e. vigorous-growing) and lower (i.e. slow-growing) nodes of soybean (*Glycine max* L.) plants using partial and whole-plant exclusion cages. In addition, we accounted for potential confounding effects of plant age on node quality by adjusting planting dates to have contrasting plant phenologies in two of the three trials reported here. By manipulating these treatments in a factorial design, we tested: 1) bottom-up effects of upper versus lower plant nodes, 2) top-down effects on upper versus lower plant nodes on aphid population growth rates, and 3) the relative strength of top-down versus bottom-up controls on aphid population growth rates. In addition, we conducted a separate field experiment to test 4) whether top-down impacts resulting from predator avoidance behaviors increase aphid dispersal.

## Materials and Methods

### Top-down versus Bottom-up Experiment

Three field trials were conducted at the University of Minnesota Outreach, Research and Education (UMore) Park near Rosemount, MN, from 19 July to 24 August 2007. In order to have plants with different ages available simultaneously, replicated plots of soybean (variety S19R5, NK®, Syngenta Crop Protection, Inc., Greensboro, NC) were established prior to experiments on 4 May, 29 May, and 26 June. Plots (10×20 m) were 8 rows wide, and planted in a randomized complete block design with one replicate of each planting date per block; ground was left fallow between plots to facilitate access. Two plant age treatments (“old” versus “young” treatments) were compared simultaneously in trials 1 and 2, whereas no young plants were available for the last trial. All plants used were at the reproductive stage (R) and were classified using the system described in Ritchie *et al*. [23]. Plants in the “old” treatments were at the beginning pod (R3, trial 2) and beginning seed (R5, trials 1 and 3) stages. “Young” plants were at the full flowering (R2, trial 2) and beginning pod (R3, trial 1) stages.

**Figure 1:**
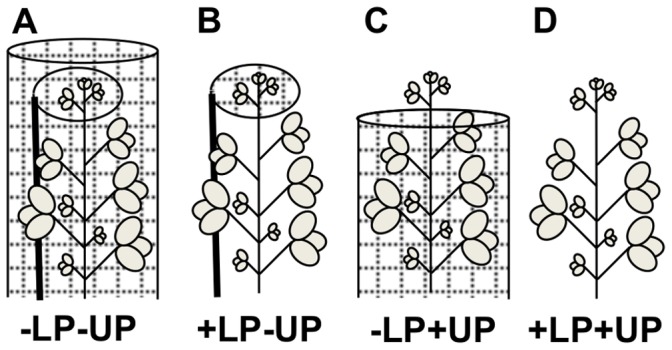
Cages used for the four manipulations of predator access to aphids at the within-plant scale. Predator treatments (LP = lower predation, UP = upper predation) corresponded to (A) predator exclusion (−LP−UP), (B) lower predation (+LP−UP), (C) upper predation (−LP+UP), and (D) ambient levels of predation (+LP+UP). The small grid pattern represents the mesh covers used to prevent aphid and natural enemy movements.

Each trial compared two levels of predation (“natural predation” versus “predator exclusion”) and locations on the plant (“lower” versus “upper” nodes), resulting in four treatment combinations: a) no predation (−LP−UP, predators excluded from the whole plant; LP = lower predation, UP = upper predation), b) lower node predation (+LP−UP, predators excluded from upper nodes), c) upper node predation (−LP+UP, predators excluded from lower nodes), and d) full predation (+LP+UP, uncaged plant allowing access to all predators) (Fig. 1). All treatments were allocated in a factorial design, with two levels of plant age×two levels of predation×two levels of within-plant location, resulting in eight treatment combinations replicated 4 times (trials 2 and 3), and four treatment combinations (only one plant age available) replicated 8 times (trial 3).

Treatments were provided using partial and total exclusion cages (Fig. 1). The whole-plant exclusion cage consisted of an internal cylindrical wire frame (tomato cages, 0.4×1.0 m, diameter×height, respectively), covered by a white, fine no-see-um netting (Kaplan Simon Co., Braintree, MA)(after [24], Fig. 1a). To assure that aphid movement between the upper and the lower nodes did not confound predator exclusion treatments, an upper predation exclusion cage was included inside the whole-plant exclusion cage. Upper exclusion cages consisted of a cylindrical mesh bag (20×30 cm) of the same material, enclosing the upper 3–4 nodes of the plant (Fig. 1b). The bag was secured to the stem with an elastic cord and supported by clipping the cage to a wooden stake. No plant damage was visible as a result of securing the bag to the stem. Lower exclusion cages were similar to whole-plant exclusion cages, but 30 cm shorter and with a small opening at the top (10 cm) that was secured to the stem by the same procedure as upper exclusion cage, enclosing the plant completely below the upper 3^rd^ or 4^th^ node, and supported externally by tomato cage frames (Fig. 1c). For the whole-plant and lower exclusion cage designs, mesh at the bottom of the cage was buried in the soil. Finally, the open treatment consisted of an individual soybean plant with neighboring plants removed (2–3 plants on each side) to minimize aphid movement between plants and provide similar growing conditions with respect to the other treatments, in which plants were removed to place cages (Fig. 1d). Lastly, we added an additional whole plant predator exclusion treatment to control for the effect of restricting aphid movement between lower and upper nodes. This treatment consisted of a whole-plant exclusion cage but without an internal upper exclusion cage, so aphids had unrestricted movement to any part of the plant. This treatment was not included in the factorial analyses of the results (see below) and was only used for comparison with the whole-plant exclusion treatment with the internal upper exclusion cage (see File S1).

These treatments were randomly applied to five plants that were selected haphazardly from the inner six rows within each plot. All resident insects were removed after careful visual inspections, and then each plant was infested with adult or nearly adult aphids (based on their size, presence of offspring around, and darker coloration of the cornicles [25]) collected from a naturally infested neighboring field. Field collected aphids did not show any evidence of parasitism during any of the trials. All plants received 10 aphids on an upper node (typically the apical new growth) and another 10 aphids on a lower node of the plant (either node six, seven, or eight, depending on plant development). Aphids were transferred using a moistened, fine camel-hair brush [25].

High *A. glycines* populations are achieved during July and August [26]; therefore, the experiments were conducted on consecutive dates starting at July 19 (trial 1), August 3 (trial 2), and August 16 (trial 3). Duration of the trials ranged from 7 (trial 3) to 14 d (trials 1 and 2), and aphid numbers on the upper and lower nodes were recorded separately each week. To account for the different lengths of each experiment, we estimated *A. glycines* growth rate using the intrinsic rate of increase, *r* = (ln(N_t_)−ln(N_0_))/t, where N_0_ = initial number of aphids, N_t_ = number of aphids at time t, and t = duration of the experiment (in days). Aphid growth rate was calculated separately for upper and lower nodes to test for within-plant bottom-up effects, and for the whole plant to contrast bottom-up and top-down effects, on overall aphid population growth.

Larger adult aphid size has been shown to be an indirect indicator of favorable plant quality and higher reproduction [14], [22], [27]. Therefore, to further test for bottom-up effects of plant quality, we contrasted the size of aphids on leaves of upper versus lower nodes, collected from cages that excluded predators (i.e. upper exclusion cages for upper nodes, lower exclusion cages for lower nodes, and whole-plant exclusion cages for both type of nodes), on August 24. Each sample consisted of all aphids present on a leaf, resulting in variable number of aphids in each sample (15.8±18.8 aphids/leaf, mean ± SD, n = 22 leaves), but precluding selection bias. Aphids were preserved in ETOH 95% until processed, for a total of 348 aphids measured from 19 plants sampled (from 3 plants we collected both upper and lower leaves and since they showed the same trends as leaves from separate plants, we considered them independent for the analysis). Abdominal widths at the widest point, and body lengths from the head to the tip of the abdomen, were measured using an ocular micrometer installed in a stereoscope microscope (Leica MZ0). Aphid size was estimated by multiplying length by width. Based on the development of the cauda, aphids were grouped into three classes, which can be used to approximate aphid developmental stages: 1) cauda absent or reduced (first and second instars), 2) cauda wider than or as wide as long (third and fourth instar), and 3) cauda longer than wide (adults) [28]. First and second instars were collected in insufficient number of samples and therefore were excluded from analysis. Bottom-up effects of leaf age on aphid size were tested separately for adult aphids and large nymphs, to minimize confounding effects of aphid developmental stage and host quality.

Finally, we compared the results of our experimental manipulations with naturally occurring aphid and predator populations from the same plots. Three to ten haphazardly selected plants were sampled in each plot at weekly intervals, counting the total number of aphids per plant (July) or estimating this count using a three node sampling unit (August) [29]. At the same time, predator populations were monitored taking four samples of 25 sweeps in each plot.

### Top-down Impacts on Aphid Dispersal among Plants

We conducted a separate field experiment to test whether exposure to predators affects the dispersal of *A. glycines* among soybean plants in a field at UMore Park, MN, during June 2008. We caged a 1-m row of soybean using a 1×1×1 m cage, which consisted of a polyvinyl chloride (PVC) frame covered primarily by no-see-um netting with a 15-cm wide band of coarser netting (2 mm) on the upper part of all cage walls to allow for alate emigration (after [30]). The netting was connected to a basal, transparent plastic barrier (10 cm buried in the soil, 20 cm above soil surface) using 2-cm wide strip of velcro. Each of 20 cages enclosed 15 soybean plants at the three to four nodes vegetative stage, with their canopies in contact, thus allowing aphids to walk among plants. On 24 June 2008 we released 50 field-collected, apterous adult aphids in the upper nodes of the five central plants (‘release plants’), at a rate of 10 aphids per plant, and allowed them to settle for 24 hr. After settling, we counted the number of aphids present on each plant and then introduced five adult, field-collected *Harmonia axyridis* Pallas (Coleoptera: Coccinellidae) in 10 randomly-selected field cages with the remaining 10 left as untreated controls. After 24 h, we recounted the number of aphids present on each plant. Since we had to remove the lady beetles from the cages to perform the aphid counts, we replaced them with freshly field-collected adult *H. axyridis* and *Coccinella septempunctata* L. (3∶2 ratio), as the latter species became more readily available. After the first 24 h of predation, we observed no impacts of the lady beetle treatment on aphid redistribution (see results), and therefore we added an additional predator treatment to the experiment by splitting the 10 control cages into two treatments. On five cages selected randomly we detached the mesh from the plastic barriers, leaving a gap of 30 cm at canopy height on all the sides. Therefore, these cages became “sham” cages, exposing aphids to ambient levels of predators (after [30]). The other five cages remained as predator-free controls. We conducted our final aphid counts 5 days later, separating “release” from “colonized” plants (i.e. sets of five plants at each side of the release plants that did not received aphids at the beginning of the experiment). We hypothesized that if predators induce escaping behaviors in the aphids, plants initially aphid-free would be colonized at higher rates in treatments exposed to predators (either the five lady beetles per cage or the ambient predator treatments), than in the predator exclusion cages.

### Statistical Analysis

To increase the power of the statistical comparisons, within-plant bottom-up effects were compared on aphid population growth rates calculated combining all the predator exclusion treatments for each plant age and trial (as plant age showed significant effects in some experiments, see results) using paired t-tests and the Satterthwaite method for unequal variances (Proc TTEST, [31]). The same procedure was used to test for the effect of aphid movement on population growth rates and proportion of aphids found on the upper nodes. Aphid size for each aphid age class was averaged per trifoliate leaf and analyzed using separate ANOVAs on square-root-transformed data. Aphid population growth rates for the whole plant and proportion of aphids on the upper nodes were analyzed using separate ANOVAs for each trial, with a split-plot model, including plant age (PA) as the whole-plot factor, and upper node predation (+UP) and lower node predation (+LP) in a factorial design as the subplot factors (Proc Mixed, [31]). Blocks were excluded from the final analysis because they were not significant. In all analyses, significant interactions were explored by slicing main effects, and means were separated using Least Square Mean Difference adjusted by the Tukey-Kramer method (LSMD-TK, [31]). In addition, since we started with equal numbers of aphids at the upper and lower nodes of the plant, we performed one sample *t*-tests to test the hypotheses that: 1) upper node predation treatments decreased the proportion of aphids on the upper nodes below 0.5; 2) lower node predation treatments increased the proportion on the upper nodes above 0.5; and 3) the combination or absence of both resulted in a proportion differing from 0.5 (P<0.05, Proc TTEST, [31]). The log_10_-transformed numbers of aphids and predators present naturally in the plots were contrasted between planting dates using one-way ANOVAs with sampling date as a repeated measures factor for the first 3 weeks of the study, and separate ANOVAs for week 5, since different planting date treatments were compared in that week (Proc GLM, [31]). Week 4 was not sampled for logistical reasons. Date and date×planting date interaction effects were adjusted for sphericity using the Huynh-Feldt test [32].

The effect of predation on aphid dispersal among plants was investigated by contrasting the total number (log_10_-transformed) and percentage (arcsine-transformed) of aphids on each of the three groups of five plants (i.e., release plants, colonized plants to the left, and colonized plants to the right) using a one-way ANOVA with predator treatment (control, lady beetles, and sham cage) as a fixed effect. Means were separated using Tukey Honest Significant Difference test (HSD, Proc GLM, [31]). These separate analyses by group of plants were carried out to avoid any potential bias of aphids moving in any particular direction from the release plants, as all plants belonged to rows similarly aligned.

## Results

### Top-down Versus Bottom-up Experiment

#### Within-plant, bottom-up effects

**Figure 2:**
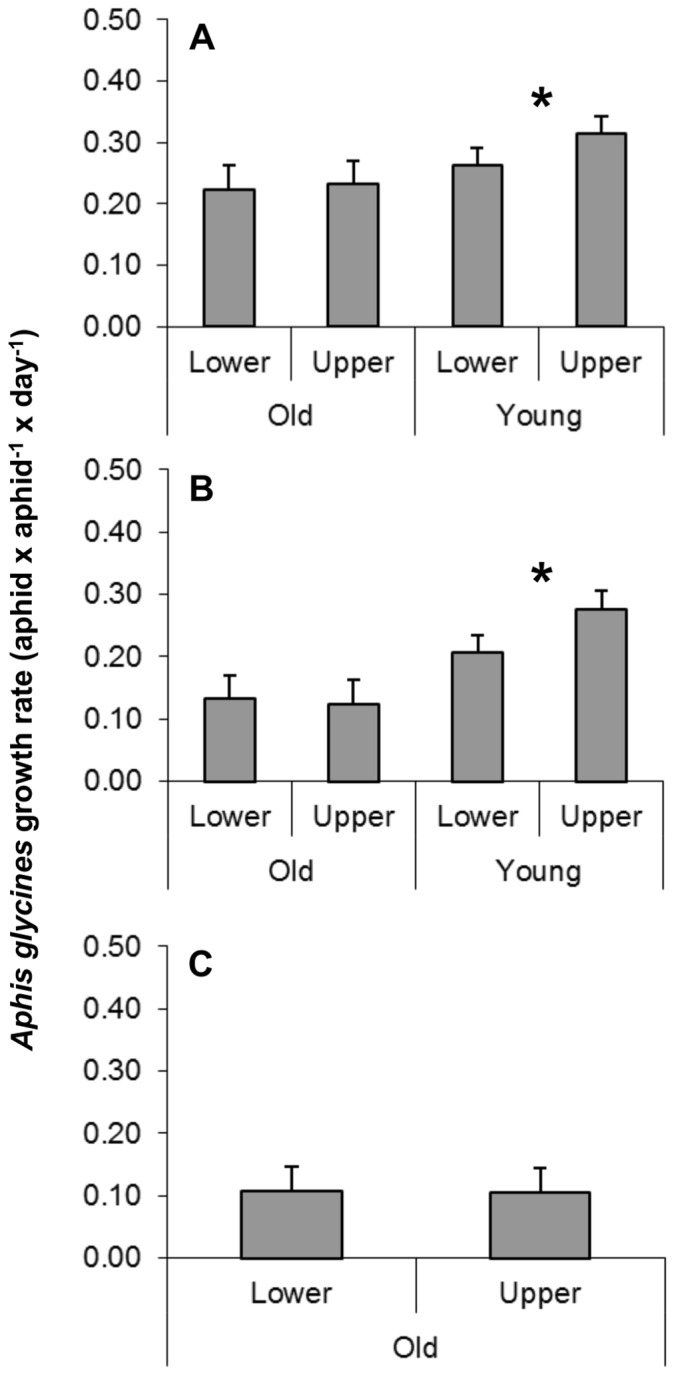
Bottom-up effects of within-plant quality on *A. glycines* population growth rates under predator exclusion. (A)–(C) present results for trials 1–3, respectively. Old versus young plants were compared in trials 1 and 2, and only old plants were available for trial 3. Upper nodes represent the 3–4 top nodes (∼ 10% of the plant canopy) and lower nodes represent the rest of the plant nodes. All graphs show means (+1 SE) of *A. glycines* intrinsic rate of increase (aphids×aphid^−1^×day^−1^); asterisks indicate significant differences between plant parts within old or young plants (*t*-test, *P*<0.05).

Combining all predator exclusion treatments, aphid population growth rates were 19.6 and 33.4% higher at the upper than the lower nodes of plants in young plant treatments for trials 1 and 2 (*t* = 3.36, df = 7, *P* = 0.012; and *t* = 3.63, df = 5, *P* = 0.015, respectively, Fig. 2a and b). By contrast, no difference in aphid growth rates was detected between upper and lower nodes in any of the old plant treatments (trial 1: *t* = 0.05, df = 3, *P* = 0.9652; trial 2: *t* = −0.21, df = 5, *P* = 0.8409; and trial 3: *t* = −0.03, df = 15, *P* = 0.9744; Fig. 2a–c).

Larger adult aphids were collected on the upper than the lower nodes (mean ± SE, 0.56±0.10 mm^2^, n = 8, and 0.28±0.04 mm^2^, n = 10; respectively, *F*_1, 16_ = 6.81; *P* = 0.0189). A similar trend was found for late instar nymphs (upper nodes: 0.26±0.08 mm^2^, n = 8; lower nodes: 0.20±0.06 mm^2^, n = 11) but differences were only marginally significant (*F*_1, 17_ = 3.85; *P* = 0.0665).

#### Top-down versus bottom-up effects on aphid within-plant distribution

Predator exclusion cages with and without restricted aphid movement did not show differences in overall aphid population growth rate or within-plant distribution, indicating absence of bias for cage manipulations on aphid dispersal between plant parts (see File S1).

**Figure 3:**
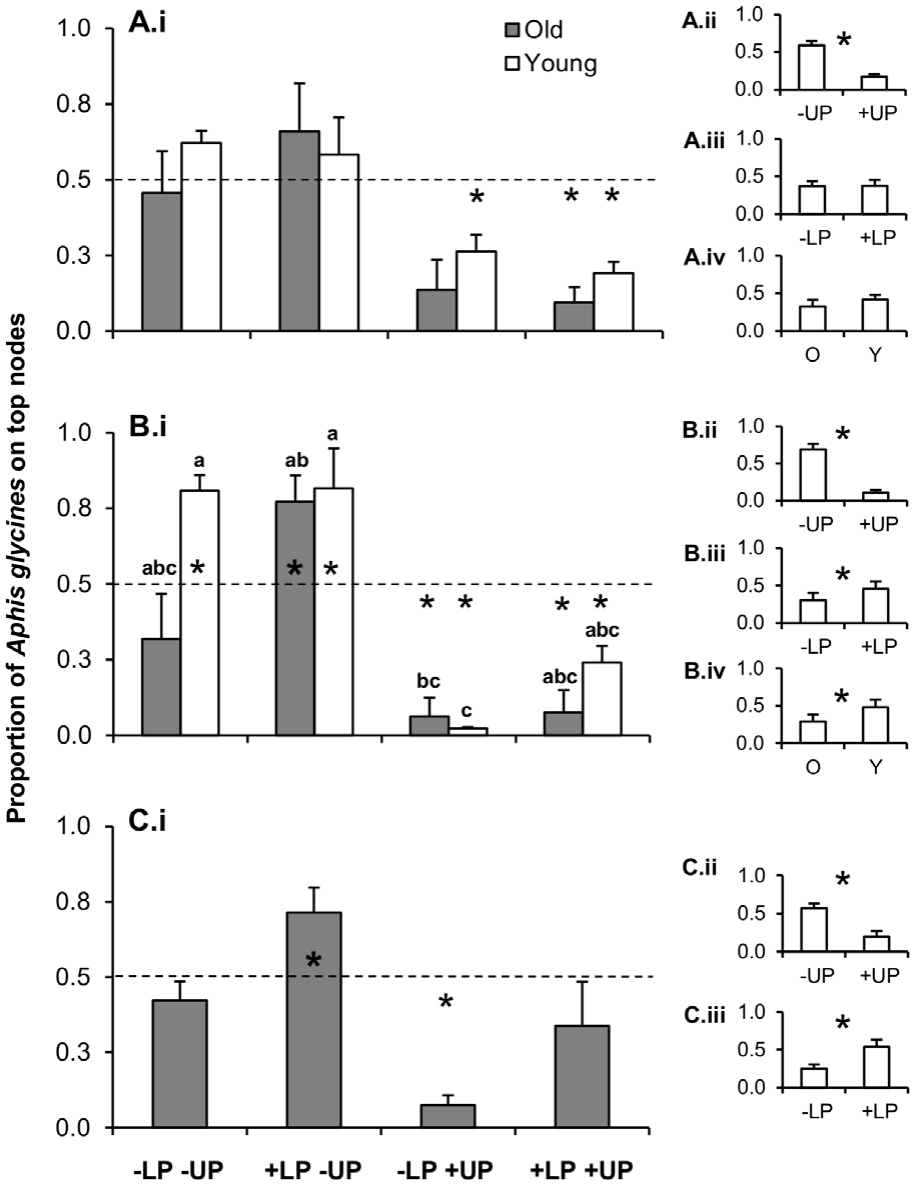
Top-down and bottom-up effects on aphid within-plant distribution. Top-down controls were manipulated as upper predation (ambient levels = +UP, or exclusion = −UP), and lower predation (ambient levels = +LP, or exclusion = −LP) (see Figure 1 for cage designs); bottom-up controls were manipulated using plants of different age (old = O, grey bars; or young = Y, white bars, plants). We present means (+1 SE) of the proportion of *A. glycines* on the upper nodes of the plants for trials 1 (A), 2 (B), and 3 (C). Means that do not share letters are significantly different (*P*<0.05, LSMD-TK tests). The dashed line indicates equal proportion at the upper and lower nodes of the plant, and asterisks above and below the line indicate significant departure from equality, testing the alternative hypothesis of different than 0.5 (controls −LP−UP, and +LP+UP), smaller than 0.5 (−LP+UP) or greater than 0.5 (+LP−UP), using *t*-tests (*P*<0.05). Small graphs (ii – iv) at the right of the main graphs (i) indicate main effects; asterisks indicate significant differences (*P*<0.05, ANOVA main effect tests, see **Table 1**).

Upper node predation significantly reduced the proportion of aphids present on the upper nodes in all three trials (Table 1). This proportion was reduced to <0.5 in eight out of ten treatments in which upper node predation was either the only source of predation or when combined with lower node predation (see *t-*test results for +UP treatments, Fig. 3). No effect of lower node predation was detected in trial 1, but it caused a small increase in the proportion on the top of the plant in trial 2, and a larger effect in the same direction in trial 3, having additive effects with upper node predation in the last two trials (Table 1, Fig. 3). In trial 2, the significant 3-way interaction indicated that lower node predation impacts were significant only in the absence of upper node predation, which was the dominant force affecting aphid distribution (Fig. 3b). Furthermore, only in the last trial did lower node predation increase the proportion of aphids on the upper nodes of the plant to levels that compensated for the shift caused by upper node predation, resulting in a proportion that did not differ significantly from 0.5 when both upper and lower node predation occurred throughout the plant (Fig. 3c).

**Table 1:** Analysis of Variance for the effects of plant age (PA), upper predation (UP), and lower predation (LP), and their interactions, on the proportion on the upper nodes of the plant and intrinsic rate of increase (aphid×aphid^−1^×day^−1^) of *A. glycines* for three field trials conducted in Minnesota, USA.

			Proportion on top		Rate of increase	
Trial	Factor	*df*	*F*	*P*	*F*	*P*
1	PA	1, 6	2.71	0.1508	**6.00**	**0.0498**
	UP	1, 11	**36.70**	**<0.0001**	**7.71**	**0.018**
	LP	1, 11	0.03	0.8613	0.14	0.7122
	PA×UP	1, 11	1.16	0.3047	0.82	0.3859
	PA×LP	1, 11	0.52	0.4874	0.17	0.6857
	UP×LP	1, 11	0.90	0.3625	2.39	0.1506
	PA×UP×LP	1, 11	0.49	0.4993	1.01	0.3356
2	PA	1, 6	**6.18**	**0.0475**	**13.65**	**0.0102**
	UP	1, 13	**73.54**	**<0.0001**	**50.62**	**<0.0001**
	LP	1, 13	**6.32**	**0.0258**	**17.63**	**0.0010**
	PA×UP	1, 13	0.02	0.9024	0.16	0.6979
	PA×LP	1, 13	0.00	0.9991	0.15	0.7019
	UP×LP	1, 13	0.00	0.9686	0.04	0.8369
	PA×UP×LP	1, 13	**6.54**	**0.0238**	4.12	0.0633
3	UP	1, 19	**22.55**	**0.0001**	**20.10**	**0.0003**
	LP	1, 19	**5.87**	**0.0256**	**7.75**	**0.0019**
	UP×LP	1, 19	0.05	0.8206	0.85	0.3690

Bottom-up effects due to differential within-plant quality also affected aphid density at different plant locations, but their impacts were strongly offset by top-down forces. Only when top-down effects were excluded was there a trend to higher proportion of aphids on the upper nodes of young plants (62.1–80.9%), with significant effects of plant age in trial 2, indicating that upper nodes had higher quality for aphid growth (Fig. 3, Table 1). In summary, upper node predation was the overriding force shaping aphid within-plant distribution, offsetting effects of plant quality and lower node predation.

#### Top-down and bottom-up effects on aphid population growth rate

**Figure 4:**
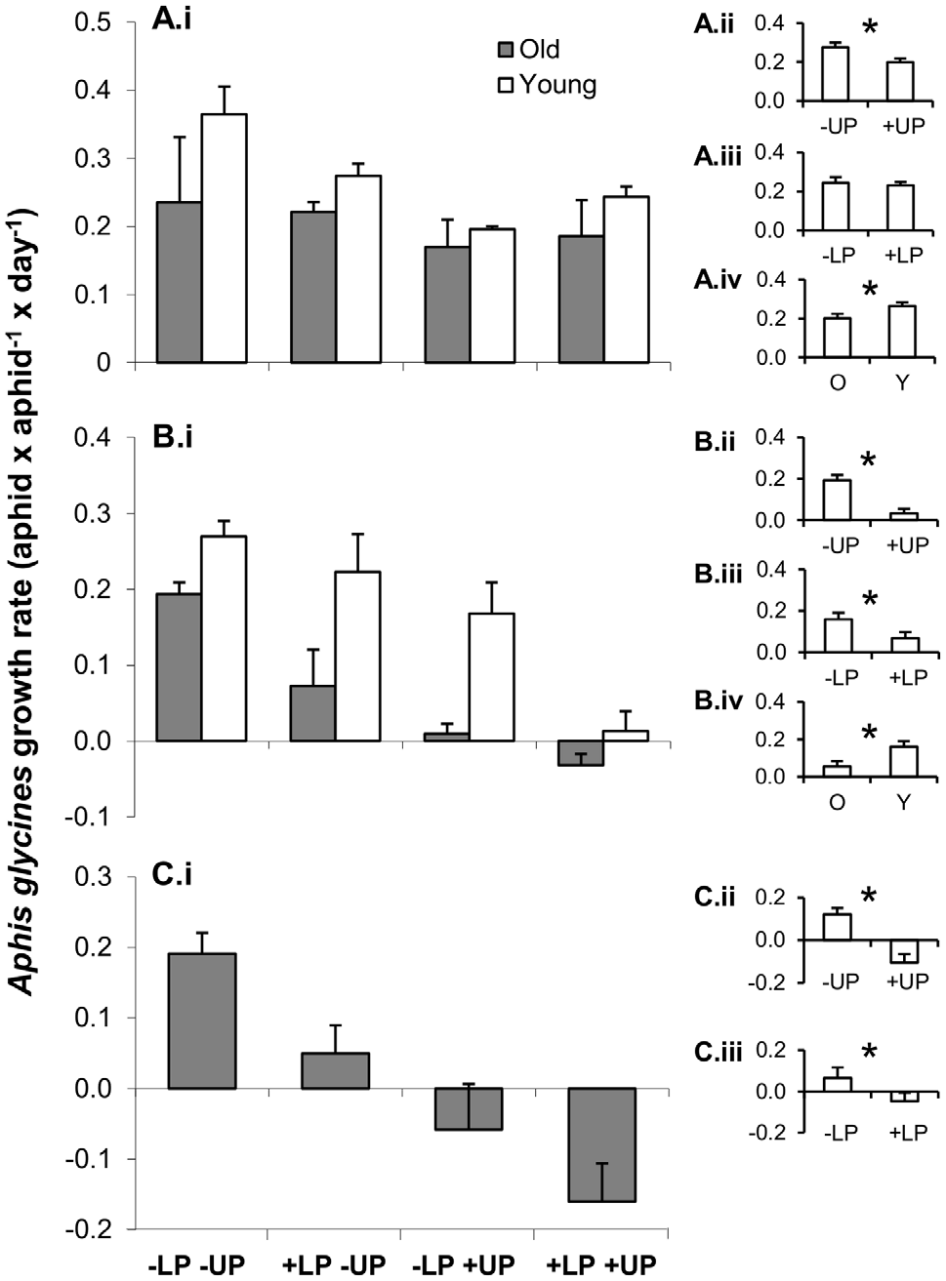
Top-down and bottom-up effects on aphid population growth rates. We present means (+1 SE) of *A. glycines* intrinsic rate of increase (aphids×aphids^−1^×day^−1^) for trials 1 (A), 2 (B), and 3 (C)**.** See Figure 3 for other references.

Upper node predation (−LP+UP) suppressed aphid populations in all trials, reducing rates of increase from 27 to 130% (67±20% mean ± SE), compared to the predator exclusion treatment (−LP−UP; Fig. 4, Table 1). Lower node predation (+LP−UP) showed smaller effects, reducing growth rates from 17 to 73% (40±11%) compared with the predator exclusion treatment, and this reduction was detected in trials 2 and 3 (Fig. 4, Table 1). Bottom-up effects of plant age also affected aphid populations, with growth rates in young plants being 37 and 67% higher than in the old plants, in trials 1 and 2, respectively (Table 1). No interactions among the main effects were detected, suggesting that all of these effects made additive contributions to population growth rates. Thus, our results suggest that top-down controls are prevalent and of higher magnitude (21 to 182% reduction growth rates) than bottom-up controls of plant age on *A. glycines* population growth. Moreover, effects of plant age that occurred without predators could not be detected with upper node predation (−LP+UP, trial 1), or were all but eliminated under full predation (+LP+UP, trials 1 and 2).

#### Aphid and predator field populations

**Figure 5:**
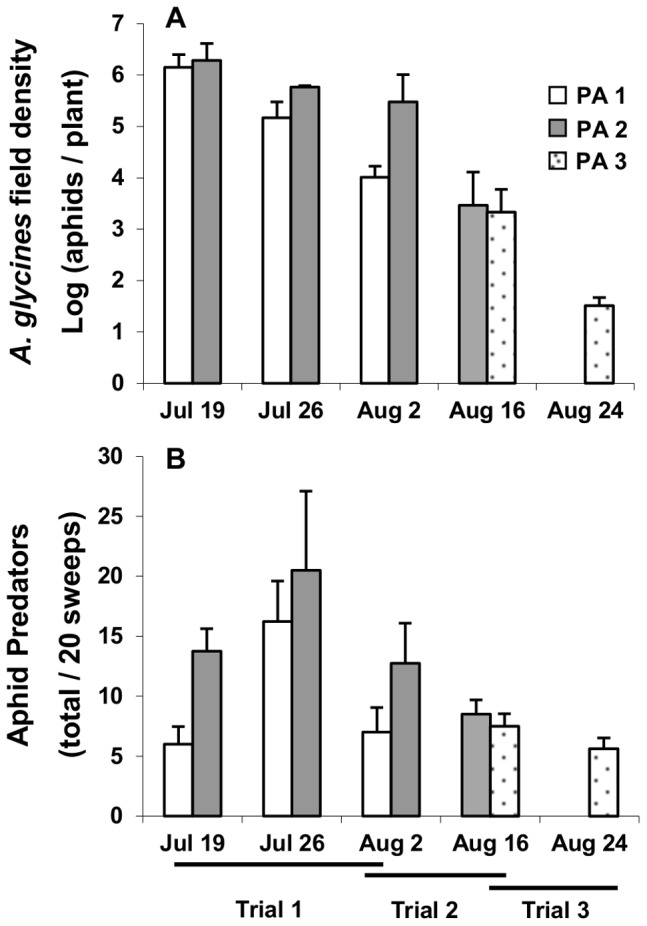
Effects of plant age (PA) on naturally occurring populations of *A. glycines* and predators in the experimental plots. Bars present mean (+1 SE) of (A) log_10_– transformed number of aphids/plant, from a sample of 10 random plants per plot in each date, and (B) total number or predators/25 sweeps, from four samples/plot. PA 1–3 refers to the oldest to the youngest plant age, respectively. Horizontal lines indicate the dates when the manipulative trials were conducted.

Naturally occurring populations of *A. glycines* varied significantly with sampling date (*F*_ 2,6_ = 8.82, *P* = 0.0163), reaching outbreak levels above the economic injury level of 674 aphids/plant during the first 5 weeks of the study (Fig. 5a). Neither plant age (PA, *F*_ 1,3_ = 1.54, *P* = 0.3026), nor PA×sampling date interaction (*F*_ 2,6_ = 0.96, *P* = 0.4335) affected aphid populations. Similarly, on 16 August planting date did not affect field population size (*F*_ 1,6_ = 0.16, *P* = 0.7052, Fig. 5a).

Predator assemblages were dominated by lady beetles (Coleoptera: Coccinellidae; *H. axyridis* 35.5%; *C. septempunctata* 11.8%, *Hippodamia convergens* Guérin-Méneville 1.9%, and *Cycloneda munda* (Say) 1%), followed by damsel bugs (Heteroptera: Nabidae; *Nabis* spp. 25.6%), spiders (Araneae 10.9%), minute pirate bugs (Heteroptera: Anthocoridae, *Orius insidiosus* (Say) 5.1%), brown lacewings (Neuroptera: Hemerobidae, 3.9%) and green lacewings (Neuroptera: Chrysopidae, 3.1%). Predator populations were significantly higher during the second week of the experiment (*F*_ 2, 12_ = 4.69, *P* = 0.0313), but after that they declined (Fig. 5b). In addition, they showed a trend of higher abundance in the young plant plots (*F*_ 1, 6_ = 4.50, *P* = 0.0781, Fig. 5b), and a non-significant PA×sampling date interaction (*F*_ 2, 12_ = 1.07, *P* = 0.3737). Plant age did not affect predator populations on 16 August (*F*_ 1, 6_ = 0.38, *P* = 0.5624, Fig. 5b).

### Top-down Impacts on Aphid Dispersal among Plants

**Figure 6:**
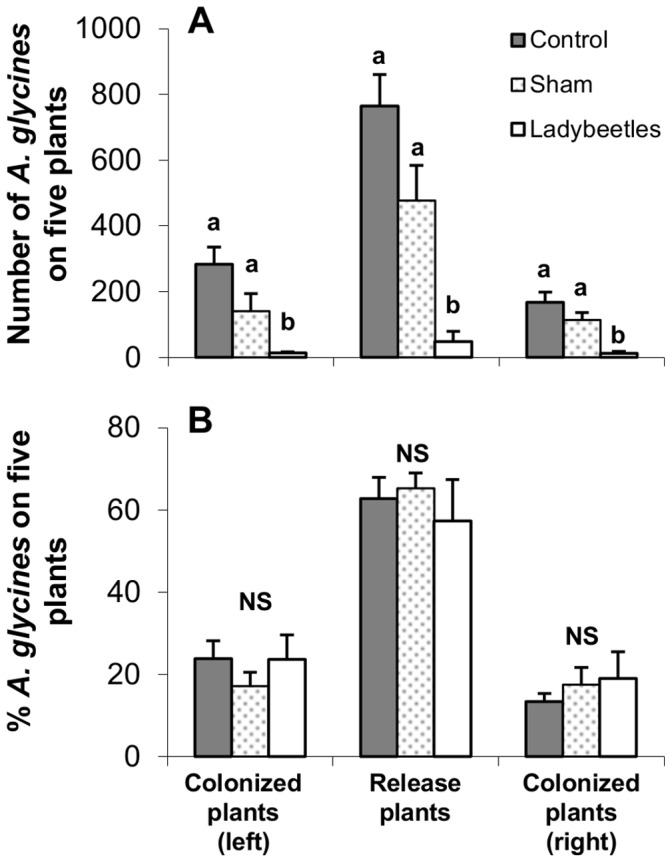
Top-down impacts on *A. glycines* dispersal among soybean plants. ( A) Number, and (B) proportion of aphids settled after five days of exposure to predation (four days for sham cages, see materials and methods for more details). Fifty aphids were released in the central 5 plants (release plants) and exposed to three treatments: lady beetles, sham and control. Lady beetle cages received five adults (*H. axyridis* and *C. septempunctata* combined) per cage, sham cages were exposed to ambient levels of all predators present in the field, and control cages had aphids only. Different letters indicate significant differences (*P*<0.05, Tukey HSD tests); NS = not significant differences (*P*>0.05, one-way ANOVAs).

Dispersal during the 24 h settling period was limited, with 85.7±2.9% of the aphids remaining on the release plants. No difference was detected in the proportion of aphids remaining on release plants between cages that later received lady beetles versus those left as controls (all *P*>0.10). During the 24 h following, predation by lady beetles was negligible: no difference in the number of aphids was detected between lady beetle treatments on the release plants (density: *F*_1,18_ = 0.13, *P* = 0.7225; proportion: *F*_1,18_ = 0.33, *P* = 0.5709); or colonized plants on the left (density: *F*_1,18_ = 0.38, *P* = 0.5451; proportion: *F*_1,18_ = 0.14, *P* = 0.7084); or right (density: *F*_1,18_ = 0.10, *P* = 0.7611; proportion: *F*_1,18_ = 0.16, *P* = 0.6898). After five more days lady beetle predation reduced aphid density by 13- to 21-fold in comparison with controls on release plants (*F*_2,16_ = 30.60, *P*<0.0001), and colonized plants (left: *F*_2,16_ = 22.60, *P*<0.0001; right: *F*_2,16_ = 18.22, *P*<0.0001; Fig. 6a). The sham treatment resulted in a trend of lower but not significantly different densities than the control (Fig. 6a). Despite the strong impact of predation observed on aphid density, the proportion of aphids present in each group of plants was unaffected (release plants: *F*_2,16_ = 0.14, *P* = 0.8687; colonized plants, left: *F*_2,16_ = 0.23, *P* = 0.7982; right: *F*_2,16_ = 0.08, *P* = 9189, Fig. 6b), supporting the conclusion that predation did not trigger *A. glycines* dispersal.

## Discussion

Our results demonstrate that a combination of top-down and bottom-up factors governs the population increase of *A. glycines*. Bottom up controls operated at the two scales investigated: between plants at different stages of development and between different-aged plant tissues on single plants. In general, we found that aphids have a higher rate of increase on younger (i.e. full flowering and beginning pod stages) than older plants (i.e. full pod and beginning seed stages), supporting predictions of the plant vigor hypothesis, and previous studies (e.g. [20], [21], [27], [33], [34]). For example, similar effects of young plant leaves have been demonstrated for *Myzus persicae* (Sulzer) and *Aphis fabae* Scopoli (Homoptera: Aphididae), resulting in a bottom-up controlled decline in field populations as the season progressed [20], [21]. At the within-plant level, we found higher rates of increase and proportions of aphids on upper nodes of young plants, whereas no differences were found between nodes of older plants, indicating that within-plant changes in quality varied with plant phenology. However, we found larger adult aphids in upper than lower nodes in the old plants used in the last trial, suggesting that young tissues had higher quality than older tissues even in old plants, although this potential for higher fecundity did not result in achieved fecundity (i.e. increased population growth) [35]. This mismatch between aphid size and population growth rate can be due to several factors, including differential allocation of resources between reproductive and somatic tissues [27], and temperature effects on aphid size [36]. The apparently higher nutritional quality of plant growing points for aphid populations has been attributed to higher amino acid concentrations [27], [37]. Thus, our results on the young plant treatments, when plants are most susceptible to outbreaking aphid populations, are consistent with the plant vigor hypothesis. The exact mechanisms operating in this system and potential effects of other phenological stages remain to be studied.

Top-down control completely counteracted bottom-up control of *A. glycines*, both at the whole- and within-plant scales, diluting differences in population growth due to plant age and reversing the within-plant relative abundance of aphids. Although *A. glycines* responded in accordance to the plant vigor hypothesis in the absence of predation, strong top-down factors exerted the most significant control of aphid populations in our experimental plants, confirming previous studies [2], [24], [25], [30], [38]–[40]. Similarly, the absence of differences in the abundance of un-manipulated aphid populations in field plots with old and young plants also suggests a dilution of bottom-up effects consistent with prevalent top-down controls. Moreover, we observed a trend of higher predator populations on the young plant treatments, suggesting that predator aggregation may have counteracted higher potential for aphid growth. In addition, previous research in this system have shown a shift of aphid within-plant distribution in response to predation on plants naturally colonized [19], indicating that the patterns observed in this study are consistent with predation under natural conditions. However, the factorial design used here allowed us to separate the effects of upper and lower predation for the first time, showing that the intensity of predation is asymmetric, with higher suppression observed on upper than lower plant nodes, overcompensating the higher rates of population growth observed on the top of the plant in the absence of predation.

The strong top-down control observed in our experimental plants was not equally effective at suppressing aphid populations naturally occurring in the field plots. Several factors can explain this mismatch, including effects of aphid immigration, and potential spill-over of predators from un-manipulated to experimental plants, resulting in artificially high predator: prey ratios. In a trial conducted two weeks before this study, the field plots received a massive immigration of alate aphids. In a separate contribution [41] we showed that this immigration completely overwhelmed top-down controls, resulting in outbreaking aphid populations. Since the objective of our study was to establish mechanisms of top-down and bottom-up regulation under usual conditions of field colonization, when predators are more likely to be effective [34], [42], [43], we started all our trials with controlled numbers of aphids in levels that mimic aphid populations under normal levels of alate immigration. Those initial levels were well within the range in which predators are effective at suppressing aphids, as observed in previous studies [2], [17], [19], [24]–[26], [30], [38]–[40], [44]–[48], whereas aphid levels in plants that received immigrant alates were beyond predator control [41].

A second hypothesis explaining the discrepancy between natural and manipulated plants is that the large number of aphids naturally occurring in our plots attracted a large number of natural enemies, resulting in artificially higher predator: prey ratios in our experimental plants and stronger top-down control in comparison with un-manipulated plants. However, several lines of evidence suggest that the strong top-down control observed in our experiment was not merely an artifact of potential “spill over” of predators. First, previous studies manipulating predation have shown strong impacts of predators on *A. glycines* in experiments conducted both during years with high [2], [19], [24] and low [24], [30] aphid populations, suggesting that top-down controls observed in experimental plots are not greatly affected by surrounding aphid densities. Second, previous studies showed an aggregative numerical response of predators to aphid density, resulting in strong density-dependent decline in aphid abundance [46], suggesting that aphids benefit from dilution effects at low densities [49]. This mechanism has also been suggested to explain the control of increasing aphid densities at the scale of patches of plants, with areas of the field with below average aphid density escaping top-down controls for shorts periods of time [19]. Therefore, although our experiments were conducted in plots experiencing high aphid densities in non-experimental plants, previous evidence on this system suggests that predator spill-over effects are not the main cause of the strong top-down control of aphid populations observed in our study.

The use of cages to manipulate natural enemies has the potential to affect micro-environmental conditions for the plants, herbivores, and predators involved in the study. In the *A. glycines* system, previous studies have utilized similar cages to demonstrate the impact of natural enemies on aphid populations [2], [17], [19], [24]–[26], [30], [38]–[40], [44]–[48]. These studies showed that cages have minimal or nil effects on temperature [25], [38], [39], [44] and relative humidity [25], comparing inside and outside cage conditions. Similarly, some studies included a “sham” cage treatment, which consisted in cages with reduced lateral openings that allow predation but have similar micro-environmental conditions as exclusion cages. These studies consistently demonstrated an absence of cage effects on the impacts of natural enemies on aphid population growth by comparing sham cages with open treatments, also exposed to predation, but with potentially different micro-environmental conditions [2], [19], [24], [25], [30], [44]. In addition, two studies showed no effects of cage treatments on soybean plant height, suggesting minimal cage effects on plant development [25], [48]. Finally, we found no differences between aphid growth rates on the upper nodes in treatments covered by single mesh (i.e. Fig 1B) versus double mesh (i.e. Fig. 1A), suggesting that mesh interference with light conditions have minimal impacts on aphid growth rates (see File S2). Although it is not possible to completely rule out any potential cage effect in our experiments, the results of previous studies and our comparisons of single versus double mesh treatments strongly suggest that the effects of bottom-up and top-down controls observed in our study are not an artifact of the caged treatments used in our manipulations.

At least two different mechanisms can explain the change in aphid within-plant relative abundance in response to predation. First, within-plant redistribution can be the result of aphid dispersal [11]. In our study, we observed a similar distribution of aphids in predator exclusion cages with restricted versus unrestricted movement, suggesting little redistribution of *A. glycines*, despite differences in quality between the top and the bottom of the plant. Previous studies on other species have shown aphid dispersion after exposure to predation [7], [11], [12]. We measured *A. glycines* relocation in response to predation at the scale of groups of plants, and did not find any response by aphids, despite strong predation by lady beetles and moderate predation in sham cages. Butler and O’Neil [50] showed defensive behaviors of *A. glycines* against *O. insidiosus*, that involved a sticky substance and a potential alarm pheromone, but the defenses resulted in very limited escape behavior. Thus, our results combined with previous studies suggest that dispersal for *A. glycines* under lady beetle predation is not an important defense.

A second mechanism that explains aphid within-plant distributions follows from patterns resulting in higher rates of predation on upper nodes of the plants. Direct field observations of predation on *A. glycines* revealed more predation on the upper-third of the plant than on the lower nodes [26]. These can be the result of predator foraging behaviors that increase predation on the top of the plant. Lady beetle larvae are negatively geotactic and positively phototactic, and adults search longer on the upper part of plants [51]. Hacker and Bertness [15] captured significantly more lady beetles on sticky traps on tall rather than on short plants, consistent with the higher levels of aphid suppression observed on tall plants in experiments with controlled aphid densities. Alternatively, even without a predator preference to search the top of the plant, the smaller leaf area of the upper nodes (∼ 10% leaf area exposed to predation) in comparison with the lower nodes (∼90%), will result in higher predator efficiency consuming aphids on the upper nodes [52]. These diverse results support the conclusion that changes in within-plant distribution of aphids are due to consumption by predators, but further research is needed to demonstrate this mechanism conclusively.

The asymmetric impact of top-down controls at the within-plant scale results in non-consumptive effects on aphid population growth that explain the strength of predator impacts on *A. glycines*. Population dynamics are significantly affected by mortality of individuals with the higher reproductive value [53]. For example, Lin and Ives [14] showed that parasitoids targeted large *A. glycines* sizes (adults or nearly adults), which presented the highest potential for aphid population increase. Using a detailed demographic model, the authors demonstrated that lower numbers of parasitoids were required to control aphid populations due to this differential removal of the individuals with the highest reproductive value. Similarly, in our study we observed the highest impact of predation on the upper nodes of the plant, which supported larger aphids and higher rates of population increase. Therefore, our results suggest that this asymmetric pressure on the top of the plant allows relatively low numbers of predators to control aphid populations. Furthermore, this non-consumptive impact of predators provide a mechanistic explanation to previous field studies that found low numbers of predators suppressing *A. glycines* populations [2], [24]–[26].

Non-consumptive impacts of predators, including trait-mediated effects on prey behavior, change of prey life history parameters, and selectivity on prey life stages [8]–[10] can have substantial impacts on herbivorous insects. Our study is one of the few that demonstrates non-consumptive impacts of top-down control through its interaction with bottom-up factors. Hacker and Bertness [15] demonstrated that predation by lady beetles on the salt marsh aphid *Uroleucon ambrosiae* (Thomas) results in almost complete exclusion of aphids from tall host plants, restricting the population to shorter plants that have low quality. Similarly, Moon and Stiling [54] showed that the planthopper *Pissonotus quadripustulatus* suffers higher impact of parasitism by *Anagrus* parasitoids on high quality green stems than on low quality woody stems. Our results, together with these previous studies, support the theoretical prediction that under strong top-down controls, herbivores will find advantage in the use of refuges, in which a trade-off of lower fecundity or longevity is compensated by a reduction of predation [19], [55]. In summary, our study adds to the growing body of literature that suggests that to correctly estimate the contribution of top-down and bottom-up controls it is necessary to study both consumptive and non-consumptive impacts of predators on herbivore populations.

## Supporting Information

File S1 **Comparison of aphid growth rates in predator exclusion cages with and without restriction on aphid movement.**
(DOC)

File S2 **Comparison of aphid growth rate in predator exclusion cages with single versus double mesh cage treatments.**
(DOC)
