# Supplementary material for: Within-Plant Bottom-Up Effects Mediate Non-Consumptive Impacts of Top-Down Control of Soybean Aphids
Source: PLoS One. 2013 Feb 19;8(2):e56394. doi: 10.1371/journal.pone.0056394 (PMC3576406; doi:10.1371/journal.pone.0056394)
Supplement: File S1 — Comparison of aphid growth rates in predator exclusion cages with and without restriction on aphid movement. (DOC) [file pone.0056394.s001.doc]

**Within-plant** **bottom-up effects mediate non-consumptive impacts of top-down control of soybean aphids**

Alejandro C. Costamagna, Brian. P. McCornack, and David W. Ragsdale

*Supporting information S1:* **Comparison of aphid GR in predator exclusion cages with and without restriction on aphid movement.**

In predator exclusion conditions, comparison of treatments with restricted versus unrestricted aphid movement did not show significant differences in population growth rate, either on old soybean plants in trial 3 (*t* = -1.37, df = 12, *P* = 0.1956, lost replicates in the unrestricted movement treatment prevented statistical comparisons in the other trials) or on young ones in trial 1 (*t* = -0.99, df = 5, *P* = 0.3662). Similarly, the proportion of aphids on the upper nodes did not differ in the old plants in trial 3 (and *t* = -1.57, df = 12, *P* = 0.1415). Similar results were found in a previous trial (population growth rate, old plants: *t* = 0.52, df = 3, *P* = 0.6389, young plants: *t* = -1.10, df = 3, *P* = 0.3531; and proportion of aphids on the upper nodes, old plants: *t* = -0.96, df = 3, *P* = 0.4073, young plants: *t* = 1.07, df = 3, *P* = 0.3641). This earlier trial had the same design as trials 1 and 2, but was affected by alate immigration and therefore we exclude it from this report, reporting the effects of immigration on predator control elsewhere [1]. The only exception to this pattern was for young plants in trial 1, where we found a significantly lower proportion of aphids on the upper nodes when movement was unrestricted (0.46 ± 0.01) than in cages restricting aphid movement (0.62 ±0.04; *t* = -4.37, df = 5, *P* = 0.0072), suggesting that aphids may have relocated to lower nodes in that particular trial. However, since the effect was relatively small and did not affect rate of increase in the same experiment, we concluded that restricting aphid movement did not bias our measurements of overall rate of increase and within-plant distribution.

1. Costamagna AC, McCornack BP, Ragsdale DW (2013) Alate immigration disrupts soybean aphid suppression by predators. Journal of Applied Entomology: in press, DOI: 10.1111/j.1439-0418.2012.01730.x.
