# Supplementary material for: Within-Plant Bottom-Up Effects Mediate Non-Consumptive Impacts of Top-Down Control of Soybean Aphids
Source: PLoS One. 2013 Feb 19;8(2):e56394. doi: 10.1371/journal.pone.0056394 (PMC3576406; doi:10.1371/journal.pone.0056394)
Supplement: File S2 — Comparison of aphid growth rate in predator exclusion cages with single versus double mesh cage treatments. (DOC) [file pone.0056394.s002.doc]

**Within-plant** **bottom-up effects mediate non-consumptive impacts of top-down control of soybean aphids**

Alejandro C. Costamagna, Brian. P. McCornack, and David W. Ragsdale

*Supporting information S2:* **Comparison of aphid growth rate in predator exclusion cages with single versus double mesh cage treatments.**

Cage treatments have the potential to affect plant photosynthesis by decreasing the light that reaches leaves. That in turn may affect aphid population growth rates. Although our design does not allow us to compare aphid growth rates in caged versus un-caged conditions due to the confounding effect of predation, we can test the effect of caging by comparing single versus double mesh cages using the upper exclusion cage (Fig. 1B) versus the whole predator exclusion cage (Fig. 1A), respectively. To test this potential cage effect, we performed separate *t*-tests on the growth rates observed on the upper nodes for each planting date and trial, and we found no significant differences between single and double mesh treatments in any of the comparisons (Table S1). Therefore, these results suggest that caging did not affect the aphid population growth rates measured in our study, suggesting that any potential effect on plant quality was also minor.

**Table S1**. Results of separate *t*-tests comparing aphid growth rates (aphid x aphid^-1^ x day^-1^) on the upper nodes of cage treatments enclosed by single (upper exclusion cage) versus double (whole exclusion cage) mesh cages.

| Trial | Plant age | Mesh | Growth rate | n | d.f. | *t* | *P* |
| --- | --- | --- | --- | --- | --- | --- | --- |
| 1 | Old | Single | 0.24 + 0.01 | 3 | 1.02* | 0.09 | 0.9395 |
|  |  | Double | 0.23 + 0.12 | 2 |  |  |  |
|  | Young | Single | 0.28 + 0.03 | 4 | 6 | -1.32 | 0.2339 |
|  |  | Double | 0.35 + 0.04 | 4 |  |  |  |
| 2 | Old | Single | 0.10 + 0.06 | 3 | 4 | -0.59 | 0.5841 |
|  |  | Double | 0.15 + 0.05 | 3 |  |  |  |
|  | Young | Single | 0.25 + 0.06 | 4 | 5 | -0.67 | 0.5321 |
|  |  | Double | 0.31 + 0.02 | 3 |  |  |  |
| 3 | Old | Single | 0.09 + 0.05 | 8 | 14 | -0.61 | 0.5486 |
|  |  | Double | 0.13 + 0.04 | 8 |  |  |  |

*Partial degrees of freedom from Satterthwaite *t*-test for unequal variances.
